# Levofloxacin loaded mesoporous silica microspheres/nano-hydroxyapatite/polyurethane composite scaffold for the treatment of chronic osteomyelitis with bone defects

**DOI:** 10.1038/srep41808

**Published:** 2017-02-02

**Authors:** Qi Wang, Cheng Chen, Wen Liu, Xiaoqiang He, Nian Zhou, Dongli Zhang, Hongchen Gu, Jidong Li, Jiaxing Jiang, Wei Huang

**Affiliations:** 1 Department of Orthopaedic Surgery, the First Affiliated Hospital of Chongqing Medical University, Chongqing, 400016, China; 2 State Key Laboratory of Oncogenes and Related Genes School of Biomedical Engineering and Med-X Research Institute Shanghai Jiao Tong University, Shanghai 200030, China; 3 Research Center for Nano-Biomaterials, Analytical & Testing Center, Sichuan University, Chengdu, 610064, China

## Abstract

Chronic osteomyelitis is a prolonged persistent disease accompanied by bone destruction and sequestrum formation, it is very difficult to treat. Antibiotic loaded polymethyl methacrylate (PMMA) has been used in clinical. However, when PMMA was implanted in the body, the deficiencies is that it is non-biodegradable and a second operation is needed. Here, we synthesize a novel levofloxacin loaded mesoporous silica microspheres/nano-hydroxyapatite/polyurethane composite scaffolds, and evaluated the therapeutic effect in treating chronic osteomyelitis with bone defects in rabbit model compared with bulk PMMA. X-ray, Micro CT, gross pathology as well as immunohistochemical staining were performed at predesignated time points (1, 3, 6 and 12 weeks). Our results demonstrated that the efficiency of mesoporous silica microspheres/nano-hydroxyapatite/polyurethane composite scaffolds loaded with 5 mg levofloxacin was much better at treating bone defects than the other groups. This novel synthetic scaffold may provide a solution for the treatment of chronic osteomyelitis.

Chronic osteomyelitis is a prolonged persistent disease caused by infection from one or several microorganisms^1^,^2^, it is accompanied by bone destruction and sequestrum formation. The rate of chronic osteomyelitis is increasing due to infections related to traffic accidents, joint replacements, and diabetes mellitus^3^,^4^. There have been improvements in the treatment of chronic osteomyelitis; however, it remains a challenge for surgeons and causes an economic burden on patients. Conventional treatment methods of chronic osteomyelitis include radical debridement of sequestrum and infected soft tissues and systemic intravenous antibiotics for 4–6 weeks. However, prolonged systemic antibiotic treatment can lead to systemic toxicity, such as nephrotoxicity or ototoxicity.

Local delivery use of antibiotics are being researched to prevent this toxicity. Klemm^5^ proposed polymethyl methacrylate as an implantation for the prevention of bone infection. Antibiotic loaded polymethyl methacrylate (PMMA) has become the most widely used drug in clinical practice, because it has lower toxicity than intravenous drugs. However, PMMA has many deficiencies– a second operation is needed when it is used for treating chronic osteomyelitis, because PMMA is non-biodegradable and therefore, it causes secondary damage to the patient. Further, the major drawbacks of PMMA are its poor ability to bind to bone tissue which hampering its biological function^6^,^7^.

Recently, many studies have focused on the use of bioabsorbable materials for treating bone infections and bone defects. Nano-hydroxyapatite (n-HA) is similar in structure to the human bone tissue structure and inorganic minerals, and has good biological activity and biocompatibility, its good biocompatibility was demonstrated by cytotoxicity assays^8^. Therefore, n-HA has been regarded as one of the most promising materials in this field. Vancomycin loaded into an n-HA composite material has been successfully used for the treatment of chronic osteomyelitis^9^. Polyurethane (PU) is another biodegradable material that has been widely used in bone tissue engineering. Polyurethane performs well in inducing bone regeneration^10^,^11^.

Mesoporous silica nanoparticles (MSNs) were first discovered by Kresge^12^. This new material possesses many advantages, including large pore volume, controllable pore size, highly specific surface area, and a modifiable surface^13^. Therefore, MSNs have been widely used in bionanotechnology and nanomedicine. A number of different drugs, and biological molecules, such as DNA or siRNA can be encapsulated in MSNs for cancer therapy and solid tumor treatment *in vitro* and *in vivo*^14^,^15^,^16^,^17^.

Levofloxacin is a group-III fluoroquinolone antibiotic that is isolated from ofloxacin^18^. Its antibacterial properties are two times higher than ofloxacin. Levofloxacin possesses broad-spectrum anti-bacterial properties and works against both gram-positive and gram-negative bacteria^19^. It has been commercially used in clinical applications for the treatment of bacterial infections of the bone, joint, respiratory system, urinary system, and skin^20^. Levofloxacin exerts antibacterial influence through inhibition of bacterial type II topoisomerase, which interferes with DNA replication and transcription^21^. It has a low molecular weight making it easy to combine with MSNs through electrostatic attraction^22^.

In this study, Levofloxacin was encapsulated in MSNs, then MSNs were combined with n-HA/PU which was used as an antibiotic delivery carrier. A novel biodegradable drug release, bioactive composite scaffold was synthesized. To our knowledge, the combination of Levofloxacin loaded MSNs with n-HA/PU for bone repair has not been reported previously. In this study we investigate the effectiveness of this new composite in the treatment of chronic osteomyelitis induced by Staphylococcus aureus in the rabbit tibia.

## Results

### Structural and morphological characterization

**Figure 1:**
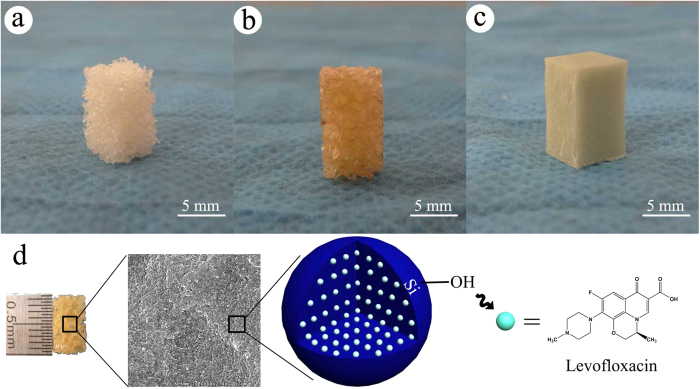
The shape and internal structure of new synthetic composite scaffolds. The n-HA/PU composite porous scaffolds were manufactured using the situ foaming method. The size of the material was 10 mm × 6 mm × 6 mm (**a**). HA/PU scaffolds were combined with MSNs, which contained different concentrations of levofloxacin (**b**). PMMA cement which contained 1 mg or 5 mg levofloxacin (**c**) was used as a positive control group. Levofloxacin was successfully loaded with mesoporous silica nanoparticle via electrostatic attraction (**d**).

**Figure 2:**
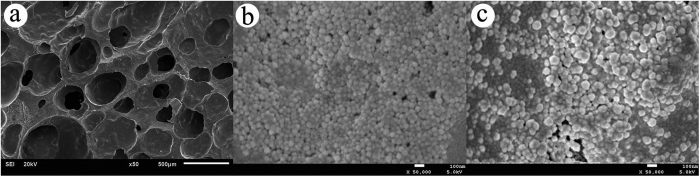
Scanning electron micrographs of the n-HA/PU scaffold (**a**), 1 mg Lev@MSNs/n-HA/PU (**b**) and 5 mg Lev@MSNs/n-HA/PU (**c**). SEM micrographs of the n-HA/PU scaffold which has many pores (**a**). It can be observed from the micrographs that there are many MSNs located on the walls of the macropores, which contain either 1 mg (**b**) or 5 mg (**c**) levofloxacin in each material.

The novel composite scaffolds were synthesized (Fig. 1). The size of the material was 10 mm × 6 mm × 6 mm. The material has a large number of pores. The average porosity of the composite scaffolds was (54.46 ± 5.68)%. n-HA/PU scaffolds are shown in Fig. 1a. n-HA/PU scaffolds were combined with MSNs, which contained different concentrations of levofloxacin (Fig. 1b). PMMA cement was used as a positive control group as shown in Fig. 1c. Levofloxacin was successfully loaded with mesoporous silica nanoparticle via electrostatic attraction, as shown in Fig. 1d. SEM micrographs of the new composites are shown in Fig. 2. n-HA was combined with PU, which has many pores and is shown in Fig. 2a. It can be observed from the micrographs that there are many micropores located on the walls of the macropores, which contain either 1 mg or 5 mg levofloxacin in each material as seen in Fig. 2b,c.

### Gross pathology

**Figure 3:**
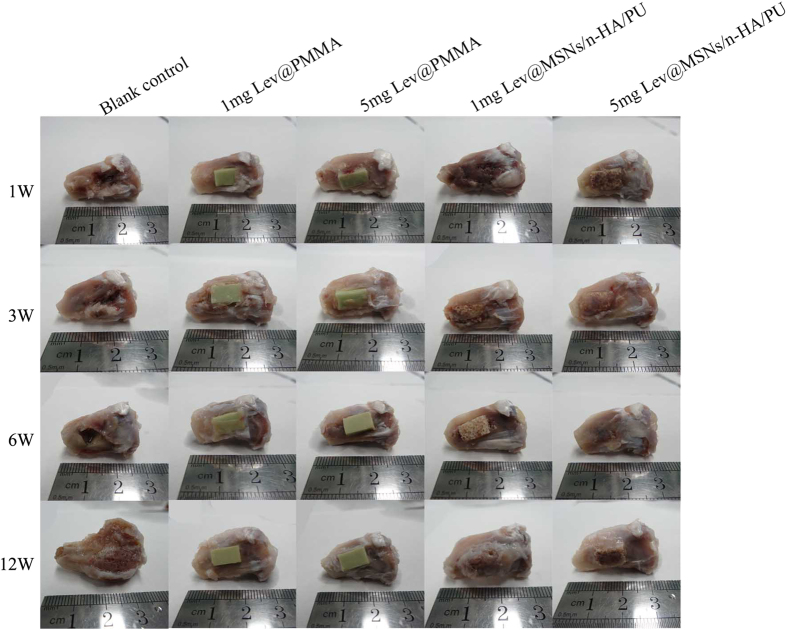
Morphological evaluation of gross pathology of each group at 1 week, 3 weeks, 6 weeks, and 12 weeks. Classical symptoms of chronic osteomyelitis such as the formation of sequestrum, bone swelling, and abscess formation could be seen in the blank control group.

The right tibia was harvested for gross pathology observation at 1 week, 3 weeks, 6 weeks, and 12 weeks Gross pathology results are shown in Fig. 3. The tibia of the blank control group had significant bone defects. The region of the bone defect increased with the increase of time after implantation. At 12 weeks, classical symptoms of chronic osteomyelitis such as sequestrum, bone swelling and abscess formation could be seen in the blank control group. However, no obvious signs of chronic osteomyelitis were seen in the other four groups. The composite was carefully combined with the tibia and was not easily removed. The structures of all of the materials remained intact.

### Radiographic evaluation

**Figure 4:**
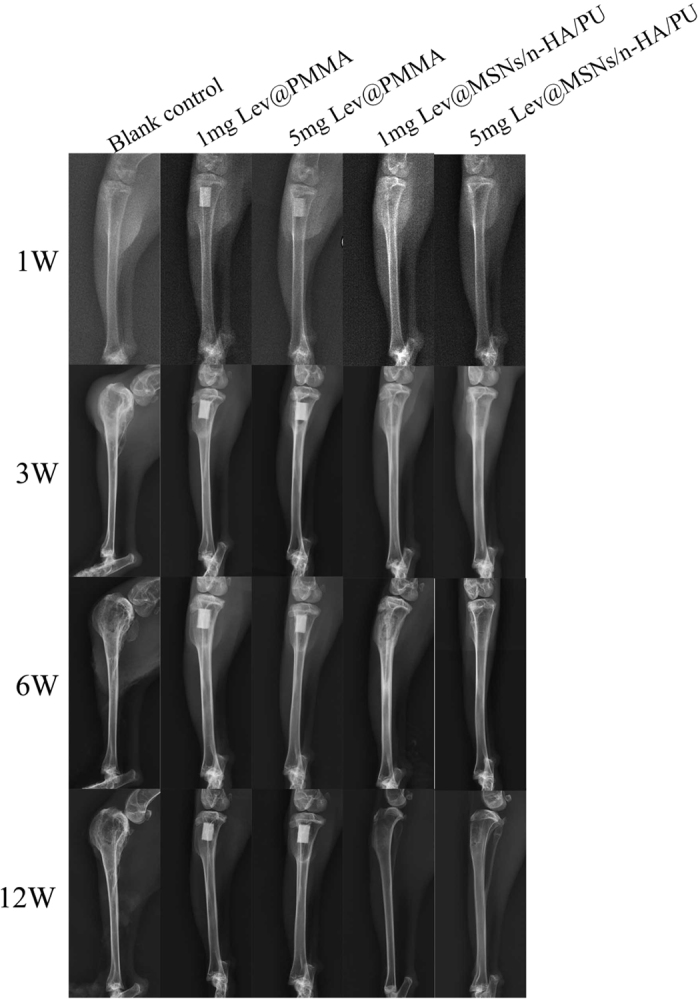
Performance of X-ray imaging in each group. At 3 week, 6 weeks, and 12 weeks, bone tissue swelling, decreased bone density, significant bone destruction, sequestrum formation, obscure boundary, osteolytic lesions, and cortical thinning were observed in the blank control group. No obvious osteomyelitis symptom were observed in the 5 mg Lev@MSNs/n-HA/PU group.

At 1 week after implantation, soft tissue swelling was clearly seen in each group. At 3 weeks, 6 weeks, and 12 weeks bone tissue swelling, decreased bone density, significant bone destruction, sequestrum formation, obscure boundary, osteolytic lesions, and cortical thinning were observed in the blank control group. Complete bone cementation was observed in both the 1 mg Lev@PMMA group and the 5 mg Lev@PMMA group. No obvious bone defects were observed in the 1mg Lev@MSNs/n-HA/PU and 5 mg Lev@MSNs/n-HA/PU group. X-ray images are shown in Fig. 4.

### Micro CT measurement

#### 3D reconstruction of the tibia

**Figure 5:**
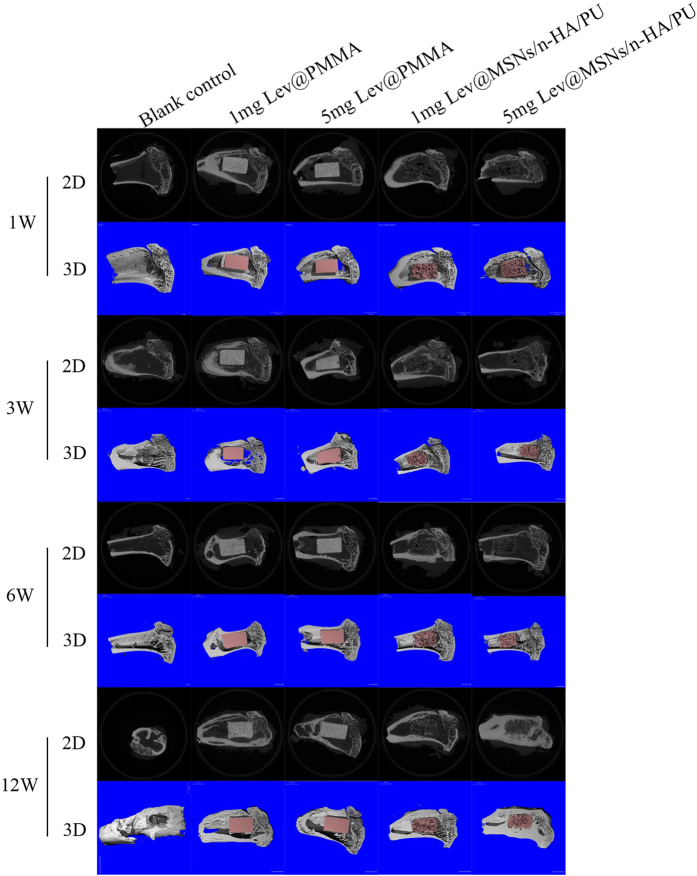
Three-dimensional reconstruction pictures of the tibia in each group at 1 week, 3 weeks, 6 weeks, and 12 weeks.

3D reconstruction images of both the tibia and the implant were observed. New bone formations around the composite scaffolds were observed at each time point. At 1 week after implantation, there was almost no new trabecular bone formation around the material. At 3 weeks after implantation, very little new trabecular bone could be observed in either the four test groups or in the blank control group. At 12 weeks after implantation 3D images of the blank control group showed typical symptoms of chronic osteomyelitis– there was obvious tibia destruction caused by inflammation, and larger areas of bone defects were seen on the 3D image. New trabecular bones were observed in the 1 mg Lev@PMMA, 5 mg Lev@PMMA, 1 mg Lev@MSNs/n-HA/PU groups. Novel trabecular bones surrounded the material and were linked closely to the material. The bone marrow cavity was filled with novel bone in the 5 mg Lev@MSNs/n-HA/PU group. 3D images are shown in Fig. 5.

#### 3D reconstruction of implant materials

**Figure 6:**
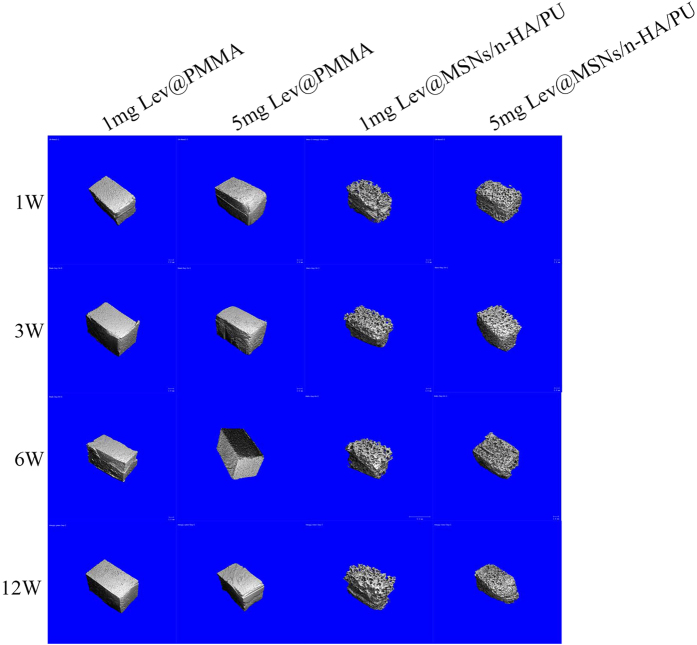
Three-dimensional reconstructions of the materials by Micro CT. PMMA maintained the integrity of its original structure at every time point; while the Lev@MSNs/n-HA/PU composite began to degrade in 12 weeks. The surface structure of the composite scaffolds appeared to collapse.

Because of the non-biodegradable characteristics of PMMA, the materials in the 1 mg Lev@PMMA and the 5 mg Lev@PMMA group maintained the integrity of their original structure at every time point. At 1 week, 3 weeks, and 6 weeks, the materials in the 1 mg Lev@MSNs/n-HA/PU and 5 mg Lev@MSNs/n-HA/PU groups maintained their original structure and didn’t show degradation. Materials in 1 mg Lev@MSNs/n-HA/PU and 5 mg Lev@MSNs/n-HA/PU groups began to degrade at 12 weeks, and the surface structure of the material appeared to collapse (Fig. 6).

#### New bone formation

**Figure 7:**
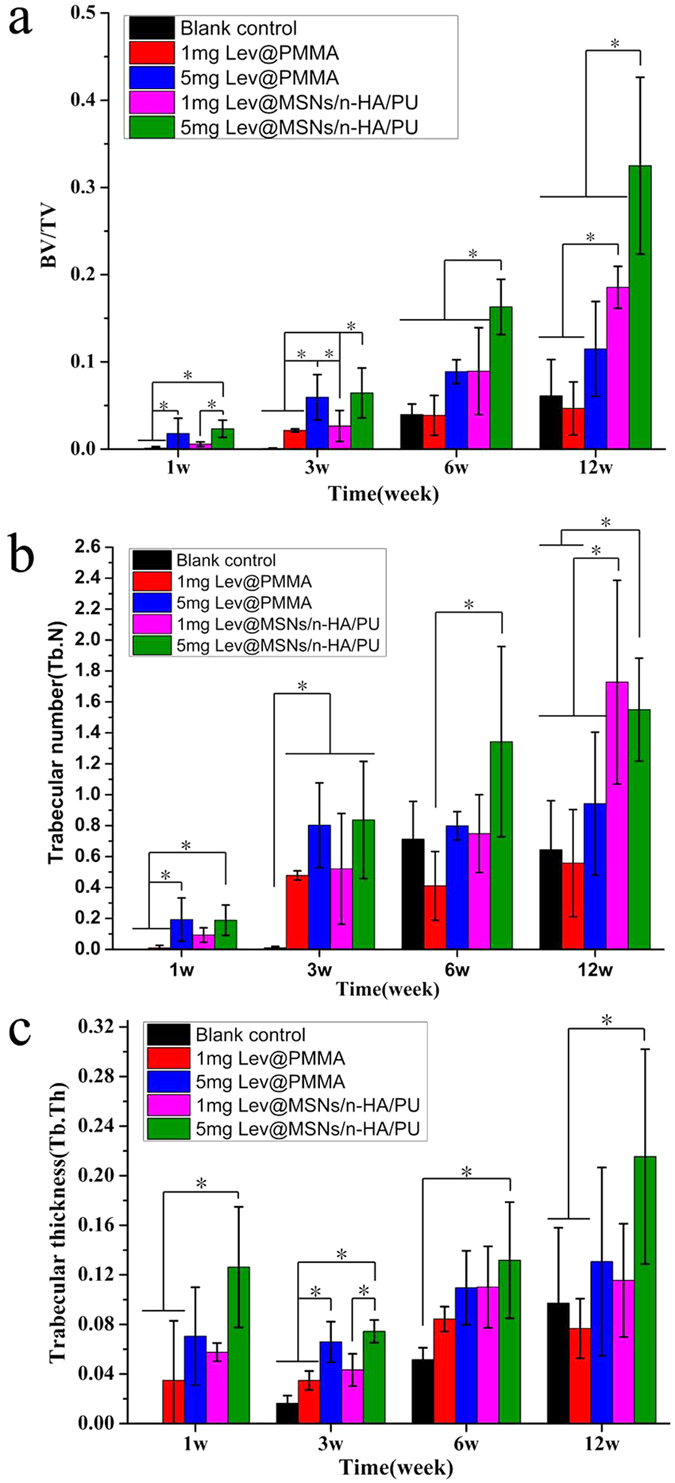
New bone formation amounts in each group at 1 week, 3 weeks, 6 weeks, 12 weeks. 3 rabbits were used in each group.

At 1 week, there was a significant differences in bone formation between the 5 mg Lev@MSNs/n-HA/PU group and the 1 mg Lev@MSNs/n-HA/PU group (*P* = 0.032), 1 mg Lev@PMMA group (*P* = 0.010) and the blank control group (*P* = 0.007). There was also significant difference between the 5 mg Lev@PMMA group and both the 1 mg Lev@PMMA (*P* = 0.041) and the blank control group (*P* = 0.031). At 3 weeks, No significant difference was found between the 5 mg Lev@MSNs/n-HA/PU group and the 5 mgLev@PMMA group (*P* = 0.737). At 6 weeks, there was a significant difference between the 5 mg Lev@MSNs/n-HA/PU group and 1 mg Lev@PMMA group (*P* = 0.000), 5 mg Lev@PMMA group (P = 0.012), 1 mg Lev@MSNs/n-HA/PU group (P = 0.013), the blank control group (*P* = 0.000). At 12 weeks, there was a significant difference between the 5 mg Lev@MSNs/n-HA/PU group and 1 mg Lev@PMMA group (*P* = 0.000), 5 mg Lev@PMMA group (*P* = 0.001), 1 mg Lev@MSNs/n-HA/PU group (*P* = 0.016), blank control group (*P* = 0.000). There was a significant difference between the 1 mg Lev@MSNs/n-HA/PU group and both the 1 mg Lev@PMMA group (*P* = 0.016) and the blank control group (*P* = 0.028). New bone formation is shown in Fig. 7a.

#### Number of new bone trabeculars

At 1 week, there was a significant difference in the number of new bone trabeculars between the 5 mg Lev@MSNs/n-HA/PU group and 1 mg Lev@PMMA (*P* = 0.014) and the blank control group (*P* = 0.010). There was a significant difference between the 5 mg Lev@PMMA group and both the 1 mg Lev@PMMA (*P* = 0.012) and the blank control group (*P* = 0.009). At 3 weeks there was a significant difference between the blank control group and 1 mg Lev@PMMA group (*P* = 0.038), 5 mg Lev@PMMA group (*P* = 0.002), 1 mg Lev@MSNs/n-HA/PU group (*P* = 0.026), 5 mg Lev@MSNs/n-HA/PU group (*P* = 0.001). At 6 weeks, there was a significant difference between the 5 mg Lev@MSNs/n-HA/PU group and the 1 mg Lev@PMMA group (*P* = 0.017). At 12 weeks, there was a significant difference between the 5 mg Lev@MSNs/n-HA/PU group and both the 1 mg Lev@PMMA (*P* = 0.015) and the blank control group (*P* = 0.024). There was also a significant difference between the 1 mg Lev@MSNs/n-HA/PU group and the blank control group (*P* = 0.009), the 1 mg Lev@PMMA group (*P* = 0.006), and the 5 mg Lev@PMMA group (*P* = 0.044). The number of new bone trabeculars is shown in Fig. 7b.

#### Thickness of new bone trabecular

At 1 week, there was a significant difference in the thickness of the new bone trabeculars between the 5 mg Lev@MSNs/n-HA/PU group and both the 1 mg Lev@PMMA group (*P* = 0.016) and the blank control group (*P* = 0.002). At 3 weeks, there was a significant difference between the 5 mg Lev@MSNs/n-HA/PU group and the blank control group (*P* = 0.001), the 1 mg Lev@PMMA group (*P* = 0.015), and the 1 mg Lev@MSNs/n-HA/PU group (*P* = 0.046). At 6 weeks, there was a significant difference between the 5 mg Lev@MSNs/n-HA/PU group and the blank control group (*P* = 0.032). At 12 weeks, there was a significant difference between the 5 mg Lev@MSNs/n-HA/PU group and both the blank control group (*P* = 0.030) and the 1 mg Lev@PMMA group (*P* = 0.014). Thickness of the new bone trabeculars is shown in Fig. 7c.

### Van Gieson staining

**Figure 8:**
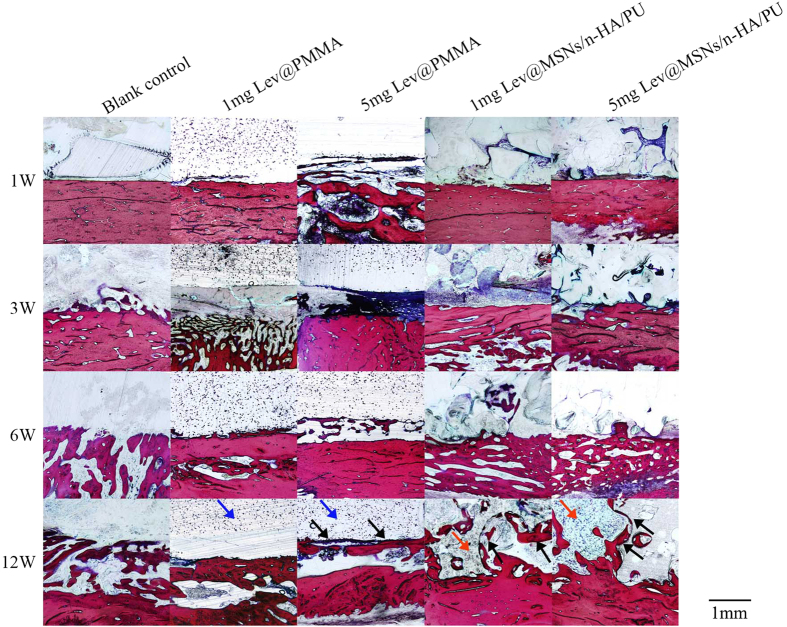
The Van Gieson staining of the tibia in each group at 1 week, 3 weeks, 6 weeks, and 12 weeks. At 1 week, there were no obviously new collagen fibers formed around the implant in all the groups. At 12 weeks, there was very little new collagen fiber formation in the blank control group. New collagen fibers grew on the surface of PMMA in both the 1 mg Lev@PMMA and 5 mg Lev@PMMA groups. New collagen fibers grew along the voids of n-HA/PU and were linked closely together in both the 1 mg Lev@MSNs/n-HA/PU and 5 mg Lev@MSNs/n-HA/PU groups. The blue arrow represents PMMA particles. The red arrow represents n-HA/PU particles. The black arrow represents new collagen fiber formation.

At 1 week, the boundary of the bone marrow cavity of the tibia was clearly seen in the blank control group. There were no obviously new collagen fibers formed around the implant in the other four groups. PMMA particles were seen in the bone marrow cavity. At 12 weeks, there was very little new collagen fiber formation in the blank control group. New collagen fibers grew on the surface of PMMA in both the 1 mg Lev@PMMA and 5 mg Lev@PMMA groups. New collagen fibers grew along the voids of n-HA/PU and were linked closely together in both the 1 mg Lev@MSNs/n-HA/PU and 5 mg Lev@MSNs/n-HA/PU groups (Fig. 8).

### Hematoxylin and Eosin staining

**Figure 9:**
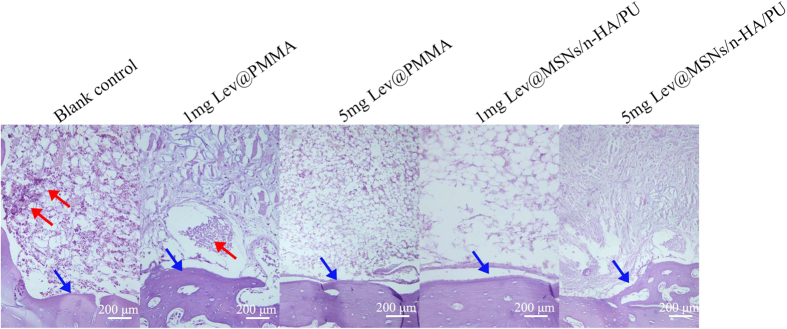
The HE staining of tibia after implantation in each group at 12 weeks (x100). A large number of inflammatory cell accumulations in the bone marrow cavity in the blank control group. Few inflammatory cells were found in the 1 mg Lev@PMMA group. The other three groups didn’t have inflammatory cells. The red arrow represents inflammatory cells. The blue arrow represents collagen fibers of the bone tissue.

The HE staining images at 12 weeks are shown in Fig. 9. In the blank control group there are a large number of inflammatory cell accumulations in the bone marrow cavity. Few inflammatory cells were found in the 1 mg Lev@PMMA group. The other three groups didn’t have inflammatory cells. This suggests that the 5 mg Lev@MSNs/n-HA/PU group has a strong ability to treat bone infection.

## Discussion

Chronic osteomyelitis is very difficult to treat, and it can have an enormous economic burden on the patient. The incidence rate of chronic osteomyelitis is increasing due to increases in traffic accidents, infections from orthopedic implants, and diabetic foot infections^2^. This presents a great challenge for surgeons, particularly when responding to infection caused by methicillin-resistant Staphylococcus aureus (MRSA). The conventional treatment method of chronic osteomyelitis is complete debridement of the sequestrum and infected soft tissue. Simultaneously, intravenous antibiotics are given. This method has the disadvantage that it may lead to large bone defects. Autologous bone graft can cause secondary injury to the patient and there are limited sources for autograft bones. Bone grafts can lead to adverse immune responses^23^. It is difficult to achieve an effective antimicrobial concentration when using intravenous antibiotics, meanwhile, systemic antibiotic treatment can lead to systemic toxicity.

PMMA cement was first used by Charnley^24^ in orthopaedic surgery. The idea to load antibiotics on PMMA for the prevention of bacterial colonization on implants was first introduced by Gartmann *et al*.^25^ in the 1970s. The infection rate of joint replacement significantly decreases when using antibiotic loaded PMMA, as compared with systematic antibiotics^26^. PMMA was chosen as a non-biodegradable local drug delivery carrier because of its lower toxicity and because it results in limited adverse reactions and few allergic reactions. Antibiotic-loaded PMMA has been commercially available in clinical settings^27^,^28^. Therefore, PMMA was selected as a positive control group in this study. However, PMMA has many disadvantages: a second operation is needed because PMMA is non-biodegradable^6^.

In recent years, biodegradable material has attracted wide attention. MSNs possess many ideal features because of their controllable pore size, high specific surface area, and large pore volume. MSNs have been widely used in bionanotechnology and nanomedicine^13^,^29^,^30^. The main pathogen leading to chronic osteomyelitis is Staphylococcus aureus (44%), Staphylococcus epidermidis (17%), streptococcus (16%)^2^. Levofloxacin is a third-generation quinolone and has antibacterial activities on both gram-positive bacteria and gram-negative bacteria^19^. Its molecular weight is small. It is easy to load antibiotics on MSNs.

Human bone is composed of inorganic compounds (65% nano-hydroxyapatite) and organic compounds (35% collagen matrix). An ideal composite scaffold should mimic natural bone. Hydroxyapatite (HA) has been used extensively because it has better osteoconductivity and biocompatibility than other biomaterials. It has been used successfully to address bone defects in clinical settings^31^,^32^. n-HA shows good performance in bone tissue engineering because of its osteogenic ability and biocompatibility^33^,^34^. n-HA is also used as an antibiotic carrier for the treatment of infection caused bone defects^9^. The applications of n-HA are limited because of its low mechanical strength and brittleness. In order to create a new synthetic material, it is necessary to composite n-HA with other biomaterials.

Polyurethane (PU) is a block copolymer consisting of isocyanates and polyester polyols^35^. Its elastic properties, thermo plasticity, mechanical strength, and biodegradability can easily be modified during PU synthesis^36^. As part of the novel scaffold, PU will be degrade into carbamic acid and polyhydric alcohols, and further through the citric acid cycle to carbon dioxide and water, PU do not have cytotoxicity, Previous study has shown that PU has been extensively applied in bone tissue engineering^37^. The bioactivity of the PU matrix increased after n-HA was introduced into it. The incorporation of 40 wt% n-HA particles in PU observably promoted new bone formation in a nude mice model^35^. Previous studies have demonstrated that n-HA can be combined with PU^38^,^39^. CS is a linear polysaccharide which consists of glucosamine and *N*-acetyl glucosamine with (1–4) glycosidic linkage^40^. The combination of CS with n-HA has been successfully used for bone tissue engineering^41^,^42^. Levofloxacin loaded MSNs were successfully combined with n-HA/PU in this experiment.

In our study, at 1 week and 3 weeks after implantation, the new bone formation of the 5 mg Lev@PMMA group and the 5 mg Lev@MSNs/n-HA/PU group are significantly different from the other three groups. However, at 6 weeks and 12 weeks after implantation, the 5 mg Lev@MSNs/n-HA/PU group has the most new bone formation.

The results suggest that antibiotics released from the composite scaffolds inhibit the process of chronic osteomyelitis soon after implantation. n-HA/PU performed better at bone repair compared with PMMA. Inflammatory cell accumulations were seen in the bone marrow cavity in the blank control and 1 mg Lev@PMMA group in the HE staining images at 12 weeks. However, the other three groups didn’t have inflammatory cells. The HE staining results were consistent with the Micro CT result. This suggests that control of the inflammation in the bone marrow cavity can result in a suitable environment for the new bone formation.

Van Gieson staining images show that new bone grew on the surface of the PMMA and along the voids of n-HA/PU, and that these growth patterns were linked closely together. This suggests that the highly porous architecture of the composite scaffolds plays a pivotal role in new bone formation. These materials provide a porous space and surface to direct cell adhesion and proliferation which is needed for new bone formation^43^.

Some *in vivo* studies have shown that there is no biodegradation of sintered HA^44^,^45^. Our study found that the novel synthetic composite scaffold began to degrade 12 weeks after implantation. Prior to 12 weeks, the integrity of the material structure provided mechanical support for bone repair, and the degradation of the biomaterial contributed to new bone formation.

## Conclusion

This study sought to emphasize on the advantages of HA while overcoming the drawbacks of n-Hap. A novel antibiotic loaded biocomposite scaffold was successfully synthesized. The therapeutic effect of 5 mg Lev@MSNs/n-HA/PU was evaluated in a rabbit model of chronic osteomyelitis. The results show that 5 mg Lev@MSNs/n-HA/PU can begin to biodegrade 12 weeks after implantation. This material has a good performance in repairing bone defects caused by chronic osteomyelitis, and controlling inflammation. In conclusion, this new antibiotic loaded biocomposite scaffold may be used as drug delivery system for the treatment of bone defects induced by chronic osteomyelitis.

## Material and Methods

### Synthesis of novel composite scaffolds

Mesoporous silica nanoparticles were manufactured according to the guidelines published in a study by Hyeon *et al*.^46^. Synthesis of Lev@MSNs used in this study was carried out according to the procedures previously reported by Gu *et al*.^22^,^47^. Cetyltrimethylammonium bromide (CTAB), sodium hydroxide (NaOH), tetraethyl orthosilicate (TEOS), and ethyl acetate were purchased from Sinopharm Chemical Reagent Co., Ltd., China. First Fe_3_O_4_ was stabilized with oleic acid. Then, the oleic acid suspension which contained Fe_3_O_4_ was placed in aqueous solution (10 mL) containing dissolved CTAB and consequently transferred to aqueous phase to form a transparent dispersion. The prepared dispersion and NaOH (2 mL) were added to water, then, heated at 70 °C for 10 minutes. Next, both ethyl acetate (2 mL, pH 1.4) and TEOS (0.5 mL) were added to the solution at 70 °C for 3 hours. Both CTAB and Fe_3_O_4_ were separated as nanocrystals when the pH value of solution decreased. Finally, levofloxacin was resolved in suspension and mesoporous silica nanoparticles were loaded by electrostatic attraction.

The steps of synthesis of Nanohydroxyapatite were as follows, the n-HA particles were prepared according to the wet chemical method without later sintering or heat treatment. A 0.3 M aqueous solution of Na_3_PO_4_·12H_2_O was added dropwise into a 0.5 M aqueous solution of Ca(NO_3_)_2_·4H_2_O at 70 °C under stirring for 1 h. The pH was adjusted to 10 with NaOH solution, and polyethylene glycol (PEG400) was employed as a surface dispersant. After reaction, the n-HA precipitate was aged for 24 h at room temperature. The slurry was freeze-dried at −50 °C for 1 week. Micrometer-sized hydroxyapatite (μ-HA) powder with a particle diameter range of approximately 5–15 μm was also fabricated by spray drying from the n-HA slurry^48^.

The n-HA/PU composite porous scaffolds were successfully manufactured using the *situ foaming method*^49^, briefly, the HA/PU composite scaffolds were fabricated by *in situ* polymerization and simultaneous foaming. First, 30 g of castor oil was mixed with 40 g of n-HA powder or n-HA particles in a 250 mL three - necked flask under nitrogen atmosphere and thorough stirring. Then, 30 g of isophorone diisocyanate was added to the mixture, and the reaction was maintained at 70 °C for 3 h to obtain the prepolymer. Subsequently, 1 mL of 1,4-butanediol was employed as a chain extender to extend the prepolymer and 0.2 mL of deionized water was added to the cross-linked prepolymer under stirring for 30 min. The resultant mixture was cured at 110 °C accompanied by simultaneous foaming. In this procedure, the HA/PU composite scaffolds were obtained. The n-HA/PU was cut into small cuboids (10 mm × 6 mm × 6 mm). First, for 20 minutes, n-HA/PU was immersed in the solution, which contained 0.5 wt% chitosan (CS) and 2% (w/w) acetic acid^50^. Then, the composite porous scaffolds were immersed into the Lev@MSNs suspension for half an hour then dried in a vacuum oven at 40 °C. Following the above steps. 1 mg levofloxacin @ mesoporous silica nanoparticle/nano-hydroxyapatite/polyurethane (1 mg Lev@MSNs/n-HA/PU) and 5 mg levofloxacin @ mesoporous silica nanoparticle/nano-hydroxyapatite/polyurethane (5 mg Lev@MSNs/n-HA/PU) were synthesized.

### Synthesis of PMMA

In this study, Polymethyl acrylate, methyl methacrylate (PMMA) was used as a positive control for the treatment of chronic osteomyelitis. Levofloxacin (Lev) (National Institute for Food and Drug Control, Bei Jing, China) was loaded on Palacos R + G bone cement (Heraeus Medical GmbH, Wehrheim, Germany). 1 mg Lev was weighed and 0.38 g PMMA powder was weighed. The Lev was uniformly mixed with the PMMA powder. The powder was mixed with 0.19 ml liquid containing methyl methacrylate. Then the mixture was stirred carefully for 30 seconds. Next, the mixture was put into the mold material (10 mm × 6 mm × 6 mm). 1 mg levofloxacin @ Polymethylmethacrylate (1 mg Lev @PMMA) was synthesized and 5 mg levofloxacin @ Polymethylmethacrylate (5 mg Lev @PMMA) was synthesized with 1 mg Lev@PMMA using the same method. All the materials were sterilized with irradiation using γ − 60 (15 KJY).

### Scanning electronic microscopy characterization of new material

After the new composite scaffolds were synthesized, the new material was coated with gold and the surface of the composite scaffolds and the morphologies of the nanosphere were observed using a scanning electron microscope (SEM, JEOL JSM-7500F, USA).

### Bacterial preparation

The standard strain of Staphylococcus aureus (ATCC 25923) was used for this study. The bacteria were placed into a sterile nutrient broth at 37 °C, and shaken overnight. The concentration of bacteria in the fresh sterile nutrient broth was adjusted to 3 × 10^7^ CFU/ml; this concentration of bacteria was calculated using the spectrophotometric standard curve. The nutrient broths containing Staphylococcus aureus were stored in a refrigerator at 4 °C.

### Animal model of chronic osteomyelitis

New Zealand White rabbits were provided by the experimental animal center of Da ping Hospital, Research Institute of Surgery Third Military University (SYXK, CQ, 2012–0006). Sixty rabbits were used in the experiment. 30 of the rabbits were male and 30 of the rabbits were female. Each rabbit weighed between 2–4 kg. All experimental protocols were approved by the Institutional Animal Care and Use Committee of Chongqing Medical University, Chongqing, China (License number: 2015–43). All the methods were carried out in accordance with the approved guidelines. The Norden method was used to create a rabbit model of chronic osteomyelitis^51^. The rabbits were anesthetized with sodium pentobarbital (1 ml/kg, body weight) and then each rabbit was fixed in the holder. The surgical site, at the right tibia, was shaved and then cleaned with povidone-iodine. Sterile surgical drapes were placed on the tibia. A 2 cm longitudinal incision was made parallel to the tibia. A 3 mm diameter hole was drilled into the medullary cavity. 0.1 ml morrhuate sodium was injected into the medullary cavity. Five minutes later, 0.1 ml of a nutrient broth that contained 3 × 10^7^ CFU/ml Staphylococcus aureus was injected. Bone wax was used to block the hole in order to prevent bacteria from leaking. During the study, all rabbits were housed individually, one per cage, and were fed a routine diet.

### Debridement and implant material

**Figure 10:**
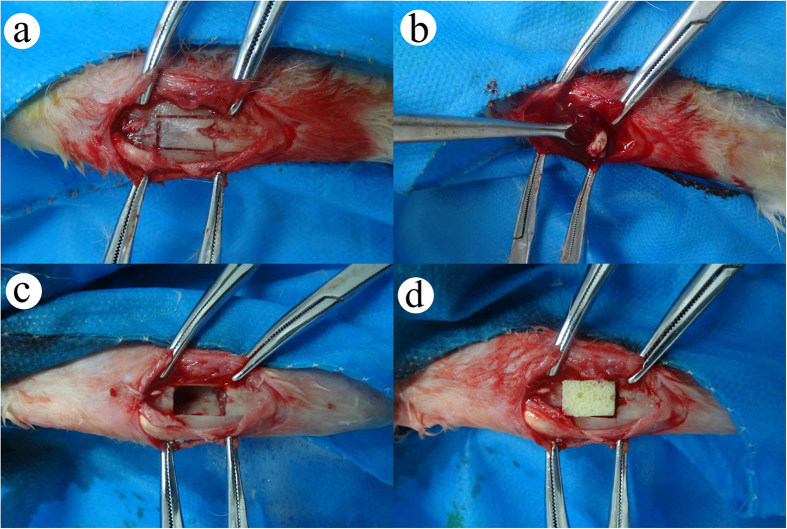
The process of material implantation. The cutting area was selected on the right side of proximal tibial (**a**). Sequestrum, inflammatory tissue and purulence was debrided by using povidone iodine and normal saline (**b**). After debridement, the size of the bone defects is 10 mm × 6 mm (**c**). The new composite scaffolds was implanted in the bone defects (**d**).

Gross pathology, pathology, and radiology were used as indices to evaluate the success of the modeling. Four weeks after infection, the model of chronic osteomyelitis was successfully established in all rabbits. All of the rabbits were treated with debridement (10 mm × 6 mm) and randomly divided into five groups. Animals in group 1 (n = 12) were only treated with debridement as a blank control. Animals in group 2 (n = 12) were treated with 1 mg Lev@PMMA. Animals in group 3 (n = 12) were treated with 5 mg Lev@PMMA. Animals in group 4 (n = 12) were treated with 1 mg Lev@MSNs/n-HA/PU. Animals in group 5 (n = 12) were treated with 5 mg Lev@MSNs/n-HA/PU. At 1, 3, 6, and 12 weeks after implantation, general observations, X-ray imaging, gross pathology, Micro CT evaluation, and histological evaluation were conducted. The surgical procedure is shown (Fig. 10).

### Radiological evaluation

X-ray imaging was used to estimate the severity of chronic osteomyelitis. The rabbits were anesthetized using an intravenous injection of 3% pentobarbital (1.0 ml/kg) and then the rabbits were fixed to the holder. Radiography was performed by X-ray machine (Carestream DRX) at four time points: 1, 3, 6, and 12 weeks after implantation. The instrument parameters were 250 mA, 44 KVP. Exposure time was 2.8 mAs.

### Gross pathology

At the four time intervals (1, 3, 6, and 12 weeks after implantation), three rabbits from each group were euthanized using an intravenous overdose of sodium pentobarbital. Soft tissue was separated. The tibia was obtained for gross bone pathology. Bone defects and formation of the sequestrum of the tibia were observed.

### Micro CT measurement

In addition to gross pathology, the three euthanized rabbits underwent Micro CT measurement. The tibia was cut into small parts and the surrounding soft tissues were removed. The tibia was soaked in 4% paraformaldehyde. The tibia sample was scanned using a Micro CT scanner (μ CT 40, Scanco Medical, Switzerland). The parameters were as follows: 300 mA, 60 KV, 1 mm thickness, and 1024 × 1024 voxel matrix. A threshold between 450 and 1000 was applied to discriminate the bone tissue, and a threshold between 220 and 450 was applied to discriminate the scaffold. Scanco medical systems (SC5073, version μ CT v6.1) were used to create 3D reconstruction images of the material. The degree of degradation of the material was observed. 2D and 3D images of the bone defects were obtained from the Micro CT scanner. The region (Voxel size: 200 × 80) closest to the material was selected as the region of interest, in order to detect new bone formation, selected areas did not contain the cortical bone. 100 CT images of each sample were used for analysis. 3 rabbits in each group each time point were measured. The same size area was used for each sample. Three quantitative indicators were assessed: bone volume to total volume ratio (BV/TV), trabecular thickness (Tb. Th), and trabecular number (Tb. N).

### Histological evaluation

After the tibia was scanned by the Micro CT scanner, the tibia was soaked in 4% paraformaldehyde for histological examination. The tibia was washed with running water for 1 hour. Each tibia was individually dehydrated with 60%, 70%, 80%, 90%, 100% alcohol for two days. The non-decalcified tibia was embedded in methylmethacrylate without decalcification for seven days. Each sample was sectioned along the longitudinal axis using a diamond cutting system (EXAKT 300 CP, Germany) at 200 μm. The slide was polished to 30 μm for Van Gieson staining (VG). The new bone formation was observed. Additionally, another tibia in each group was decalcified with 4% HNO_3_ and was dehydrated with a sequentially increasing concentration of ethanol. The slide was sectioned at 5 μm using a Leica RM2235 microtome (Leica Microsystems, Germany) for hematoxylin and eosin (HE) staining. Inflammatory cells of the bone marrow were observed.

### Statistical analysis

All quantitative data are presented as mean ± standard deviations. Significant differences were analyzed using one-way ANOVA tests using SPSS version 19.0 (SPSS, Chicago, IL USA). Statistical significance was set at *P* < 0.05.

## Additional Information

**How to cite this article**: Wang, Q. *et al*. Levofloxacin loaded mesoporous silica microspheres/nano-hydroxyapatite/polyurethane composite scaffold for the treatment of chronic osteomyelitis with bone defects. *Sci. Rep.* **7**, 41808; doi: 10.1038/srep41808 (2017).

**Publisher's note:** Springer Nature remains neutral with regard to jurisdictional claims in published maps and institutional affiliations.
